# Is multidetector CT-based bone mineral density and quantitative bone microstructure assessment at the spine still feasible using ultra-low tube current and sparse sampling?

**DOI:** 10.1007/s00330-017-4904-y

**Published:** 2017-06-21

**Authors:** Kai Mei, Felix K. Kopp, Rolf Bippus, Thomas Köhler, Benedikt J. Schwaiger, Alexandra S. Gersing, Andreas Fehringer, Andreas Sauter, Daniela Münzel, Franz Pfeiffer, Ernst J. Rummeny, Jan S. Kirschke, Peter B. Noël, Thomas Baum

**Affiliations:** 1 Department of Diagnostic and Interventional Radiology, Klinikum rechts der Isar, Technische Universität München, Ismaninger Str. 22, 81675 Munich, Germany; 2 Philips GmbH Innovative Technologies, Research Laboratories, Hamburg, Germany; 3 TUM Institute for Advanced Studies, Technische Universität München, Garching b. Munich, Germany; 4 Lehrstuhl für Biomedizinische Physik, Technische Universität München, Garching b. Munich, Germany; 5 Section of Diagnostic and Interventional Neuroradiology, Klinikum rechts der Isar, Technische Universität München, Munich, Germany

**Keywords:** Computed tomography, Radiation dose, Sparse sampling, Osteoporosis, Trabecular microstructure

## Abstract

**Objective:**

Osteoporosis diagnosis using multidetector CT (MDCT) is limited to relatively high radiation exposure. We investigated the effect of simulated ultra-low-dose protocols on in-vivo bone mineral density (BMD) and quantitative trabecular bone assessment.

**Materials and methods:**

Institutional review board approval was obtained. Twelve subjects with osteoporotic vertebral fractures and 12 age- and gender-matched controls undergoing routine thoracic and abdominal MDCT were included (average effective dose: 10 mSv). Ultra-low radiation examinations were achieved by simulating lower tube currents and sparse samplings at 50%, 25% and 10% of the original dose. BMD and trabecular bone parameters were extracted in T10–L5.

**Results:**

Except for BMD measurements in sparse sampling data, absolute values of all parameters derived from ultra-low-dose data were significantly different from those derived from original dose images (p<0.05). BMD, apparent bone fraction and trabecular thickness were still consistently lower in subjects with than in those without fractures (p<0.05).

**Conclusion:**

In ultra-low-dose scans, BMD and microstructure parameters were able to differentiate subjects with and without vertebral fractures, suggesting osteoporosis diagnosis is feasible. However, absolute values differed from original values. BMD from sparse sampling appeared to be more robust. This dose-dependency of parameters should be considered for future clinical use.

***Key Points*:**

*• BMD and quantitative bone parameters are assessable in ultra-low-dose in vivo MDCT scans.*

*• Bone mineral density does not change significantly when sparse sampling is applied.*

*• Quantitative trabecular bone microstructure measurements are sensitive to dose reduction.*

*• Osteoporosis subjects could be differentiated even at 10% of original dose.*

*• Radiation exposure should be considered when comparing quantitative bone parameters.*

**Electronic supplementary material:**

The online version of this article (doi:10.1007/s00330-017-4904-y) contains supplementary material, which is available to authorized users.

## Introduction

Osteoporosis is a skeletal disorder, leading to an increased risk for fragility fractures [1]. The loss of bone mineral density (BMD) and the deterioration of bone microstructure is typically unnoticed until actual fractures occur. Osteoporotic fractures do not only reduce quality of life, but are also associated with increased mortality [2]. The prevalence of osteoporotic fractures is increasing in our aging society and causing a large burden to healthcare systems [3]. In order to prevent the occurrence of osteoporotic fractures, it is of great importance that patients at risk are identified in order to receive proper treatment in a timely manner.

The current clinical standard method to measure BMD is via dual-energy X-ray absorptiometry (DXA). However, it is partly insufficient in identifying subjects at high risk for osteoporotic fractures: Studies have shown that over half of all non-vertebral fractures occurred in patients with non-pathological BMD values [4, 5]. Quantitative BMD and trabecular bone microstructure analysis based on high-resolution multidetector computer tomography (MDCT) improves the prediction of biomechanical bone strength and fracture risk beyond BMD [6–8]. However, the currently applied radiation dose is clinically not acceptable, particularly for longitudinal assessment of fracture risk and therapy monitoring [9].

Several parameters contribute to the radiation dose of a conventional MDCT, namely tube current, peak kilovoltage (kVp), pitch and gantry cycle time [10]. In this study we considered two approaches to reduce the radiation dose. The first approach was to reduce the X-ray tube current, which can currently be realized with a commercial scanner. Due to the nature of commercially available detectors, lowering the tube current linearly decreases radiation dose, but causes a much lowered signal-to-noise ratio (SNR) at the detector, which reduces the diagnostic quality of the images. The second approach was to acquire fewer projections during the scan, which we refer to as sparse sampling. With the future development of X-ray generator units, it will be possible to switch off the X-ray source in MDCT scanners at certain angles and provide shorter X-ray pulses, thus keeping the SNR but lowering the total radiation dose in practice.

With a reduced radiation dose, it is still possible to generate images with relatively high diagnostic quality by applying advanced reconstruction algorithms, e.g. statistical iterative reconstruction (SIR). The performance of SIR under lower tube current [11–15] or fewer projection angles [16] has been validated. To reduce the resulting noise and artefacts, SIR uses a regularization term to produce smoother images.

The aims of our study were: (i) to assess the feasibility of BMD and trabecular bone microstructure parameter measurements in MDCT examinations when tube current is ultra-low or data is sparsely sampled, (ii) to compare their values with those derived from the original imaging data, and (iii) to investigate the possibility of differentiating subjects with and without prevalent vertebral fractures with the acquired quantitative measurements.

## Materials and methods

### Subjects and multidetector CT (MDCT) scanning

Institutional review board approval was obtained for this retrospective study. Twelve subjects with osteoporotic vertebral fractures and 12 age- and gender-matched subjects without fractures who all underwent routine thoracic and abdominal MDCT were retrospectively identified and included in this study. The presence of actual fractures was determined by radiologists. Collected scans had no original purpose for osteoporotic screening.

MDCT scans were acquired using a 256-row scanner (iCT, Philips Healthcare, Best, The Netherlands). Clinical thoracic/abdominal protocols after standardized intravenous contrast agent application were used in all subjects. For calibration purposes, a reference phantom (Mindways Osteoporosis Phantom, Austin, TX, USA [17]) was placed in the scanner mat beneath the subjects. The helical pitch was set as 0.91 in 18 subjects and 0.75 in four subjects. The tube voltage was 120 kVp in all cases. Maximum tube currents of the 24 scans ranged between 200 mA and 400 mA, while exact tube current during each scan was modulated implicitly by the scanner. The average of the exposure values recorded in all 24 dose reports was 109 mAs (min: 33 mAs, max: 188 mAs).

### Tube current simulation and sparse sampling

For all subjects, we used a simulation tool to generate lower tube current scans. The simulation algorithm was based on raw projection data, as described in detail previously [18]. System parameters of the CT scanner, such as detector gain, were taken into consideration to properly account for electronic noise. Therefore, the result was accurate especially for ultra-low tube current [19]. Low-dose simulations at 50%, 25% and 10% of the original tube current were generated.

Sparse sampling was applied at levels of 50%, 25% and 10% of the original projection data. This was done by only reading every second, fourth and tenth projection angle and deleting the remaining projections in the sinogram. Thus, only the number of projections per full rotation was reduced, whereas other parameters, such as projection geometry and patient location, were kept the same.

Table 1 provides the average radiation exposure of all scans in this study. Original exposure (mAs) and CT Dose Index (CTDI_vol_, mGy) were extracted from the radiation dose report. Effective radiation dose (mSv) was approximated [28] for a female shoulder to middle thigh. Both sparse sampling and lowering tube current reduced the radiation dose linearly.

**Table 1:** Mean radiation exposure, estimated effective dose, signal-to-noise ratio (SNR) and contrast-to-noise ratio (CNR) of images under different simulated levels of the original scan

	Mean exposure (mAs)	Mean CDTI_Vol_(mGy)	Effective dose (mSv)	SNR	CNR
Original	109	7.5	10	32.802	13.682
Proj50	55	3.8	5	21.483	8.597
Proj25	27	1.9	3	16.193	6.336
Proj10	11	0.8	1	14.208	5.619
Tube50	55	3.8	5	18.552	7.234
Tube25	27	1.9	3	9.524	3.794
Tube10	11	0.8	1	2.858	1.146

Proj50, Proj25 and Proj10 represent 50%, 25% and 10% of the sparse sampling, respectively

Tube50, Tube25 and Tube10 represent 50%, 25% and 10% of the simulated lower tube current, respectively

Effective dose was estimated for a female from the shoulder to the middle of the thigh

SNR was estimated within a homogenous region of the material of the highest density in the phantom

CNR was estimated between the homogenous regions of the materials of the highest and lowest density in the phantom

### Statistical iterative reconstruction

Collected subjects were all reconstructed anew with statistical iterative reconstruction (SIR) in a much denser grid focusing on the spine. SIR was applied on the full dose data, the simulated projection data at 50%, 25% and 10% as well as the sparse sampling data at levels of 50%, 25% and 10%. SIR was performed with ordered-subset separable paraboloidal surrogate [20] combining a momentum-based accelerating approach [21].

The objective function of SIR consisted of a likelihood term and a regularization term. The likelihood term was computed with log-converted projection data. A Gaussian noise model was applied. We assumed the reconstruction with a helical path can converge with the selected solver. The iterative reconstruction stopped at a manually given number of iterations. Each small update of image *u* was made with a subset of projection data. This required one forward projection and one back projection. The forward and back projectors were implemented with a highly parallel framework [22].

For a non-sparse-sampling scan, the scanner acquired 2,400 projections per full rotation (PPR) and we chose a subset size of 100 projection images in one full rotation. The total number of iterations was 15, which yielded 360 subset updates. For 50%, 25% and 10% sparse sampling data, we kept the subset size as 100 projection images per PPR and increased the iteration number to be 30, 60 and 150, respectively. The selection of projection images was made randomly for each subset.

Regularization was applied between updates of the subsets. The regularization term was based on a Huber penalty. In order to preserve the trabecular bone microstructure as much as possible, regularization with a limited influence was selected [23].

All image slices were reconstructed as 1,152 × 1,152 pixels with a field of view of 450 × 450 mm^2^. All parts of the patient and the table were included in the field of view. Reconstruction slice interval was 0.3 mm. The resulting images had voxel spacing of 0.39, 0.39 and 0.3 mm in three dimensions. The exact voxel size was limited to the collimator width at the detector. Voxel intensities (linear attenuation coefficients) were translated to Hounsfield units (HU) using air/water information from the calibration of the scanner. Patient position and table height were incorporated so that all images for each individual were automatically registered.

### Bone mineral density and trabecular microstructure analysis

Hounsfield units inside the vertebrae were calibrated to BMD values by using the reference phantom [17]. The phantom consisted of five rods of basic materials with known equivalent water and K_2_HPO_4_ densities. Transferring coefficients were thus calculated in a least squares manner with all five rods for each subject independently. To eliminate the effect of the intravascular contrast agent, a conversion equation was applied empirically as described previously [24].

Mean BMD and standard deviation were calculated inside regions of interest (ROIs) within the trabecular part of the vertebral bodies of each subject. ROIs were placed inside the ventral part, within the central third of the height of the thoracic vertebrae T10–T12 and lumbar vertebrae L1–L5 similar to standard QCT measurements. All ROIs were cylinders with a diameter of 14–16 mm and a height of 4–6 mm, depending on the individual’s vertebrae size. The same ROIs were applied for original dose images, sparsely-sampled and simulated lower-current images. Vertebrae with fractures were excluded from the ROI selection.

Trabecular bone microstructure within the ROIs was analysed with an in-house developed program based on IDL (Interactive Display Language; ITT Visual Information Solutions, Boulder, CO, USA). Similar to previous studies [25], voxels in ROIs were binarized to be either bone or marrow. A global threshold was chosen as 200 mg/cm^3^ equivalent K_2_HPO_4_, which was optimized visually for the microstructure analysis of the subjects [26]. Four morphometric parameters were estimated: bone volume over total volume as bone fraction (BF), trabecular number (TbN, mm^-1^), trabecular separation (TbSp, mm) and trabecular thickness (TbTh, mm) [9]. Parameters were labeled as apparent (app.) due to the limited spatial resolution. One texture parameter of the trabecular bone microstructure (fractal dimension, FD) was determined using a box counting algorithm [27].

### Statistical analysis

The statistical analysis was performed with SPSS software package (SPSS, Chicago, IL, USA). All tests were done using a two-sided 0.05 level of significance. Mean and standard deviation of BMD and trabecular bone microstructure parameters were calculated. The Kolmogorov-Smirnov test showed for most parameters no significant difference from normal distribution (*p* < 0.05). Therefore, paired t-tests were used to evaluate the differences between BMD and trabecular bone microstructure parameters derived from the different sparse sampled and low-dose simulated data in all subjects. Differences in BMD and trabecular bone microstructure parameters derived from the different sparse sampled and low-dose simulated data between the matched groups of subjects with and without osteoporotic vertebral fractures were evaluated with paired tests and area under the receiver-operating characteristic (ROC) curves.

## Results

**Fig. 1:**
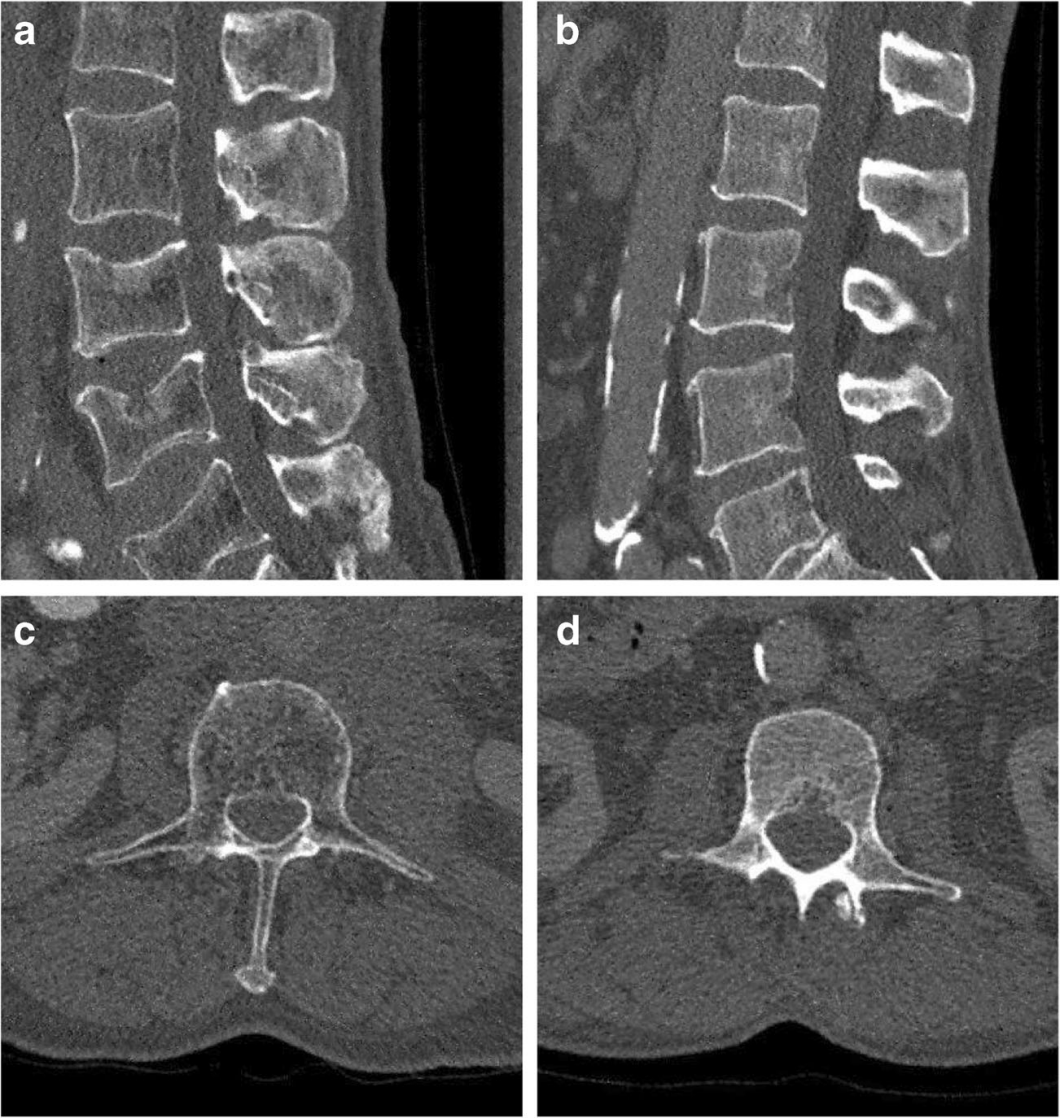
Representative reconstructions of the lumbar spine (L1–L5) of in-vivo spine multidetector CT (MDCT) data at the original dose. The left column depicts a subject with a fracture; the right column displays the matched healthy subject with regard to age and gender. The original dose was at 120 kV, 107 mAs (left) and 114 mAs (right) (exact tube current was modulated). Window level was 300 HU and width was 1,500 HU. Field of view was 180 × 153 mm^2^ for (**a**) and (**b**), and 156 × 156 mm^2^ for (**c**) and (**d**)

**Fig. 2:**
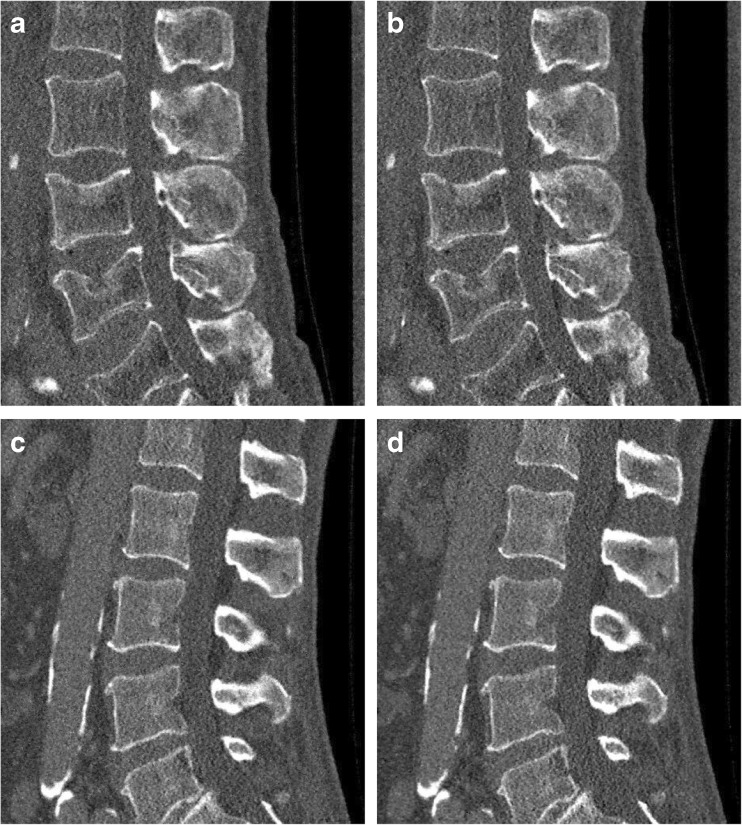
Representative sagittal reconstructions at 50% of the original dose level. The images depict the corresponding results for Fig. 1a and Fig. 1b. (**a**) and (**c**) represent the statistical iterative reconstruction (SIR) reconstructed image with 50% sparsely sampled projection. (**b**) and (**d**) show the SIR reconstructed images with the simulated 50% of the original tube current. Window level was 300 HU and width was 1,500 HU. Field of view was 180 × 153 mm^2^

**Fig. 3:**
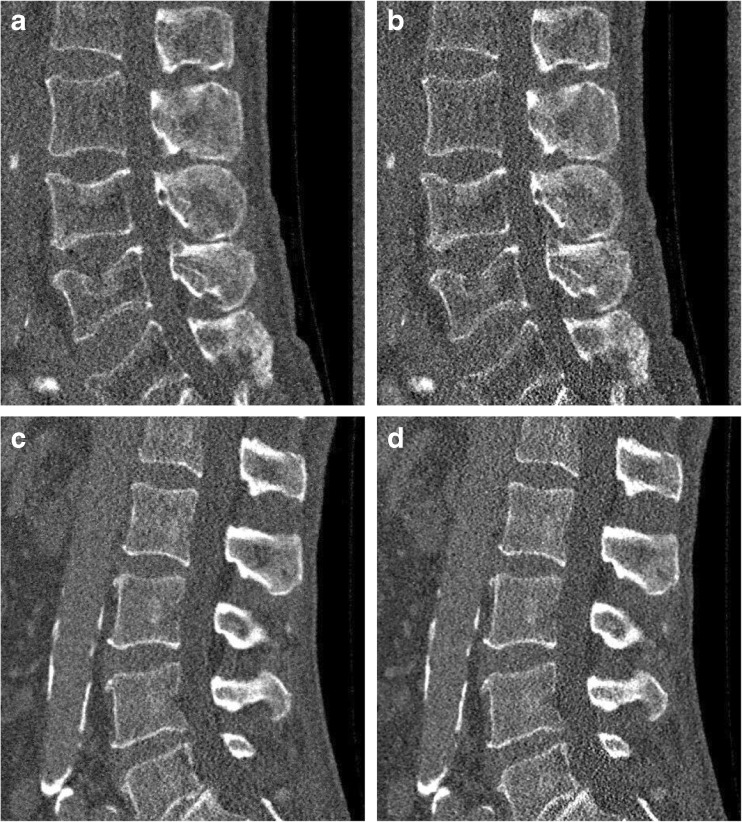
Representative sagittal reconstructions at 25% of the original dose level. The images depict the corresponding results for Fig. 1a and Fig. 1b. (**a**) and (**c**) represent the statistical iterative reconstruction (SIR) reconstructed image with 25% sparsely sampled projection. (**b**) and (**d**) show the SIR reconstructed images with the simulated 25% of the original tube current. Window level was 300 HU and width was 1,500 HU. Field of view was 180 × 153 mm^2^

**Fig. 4:**
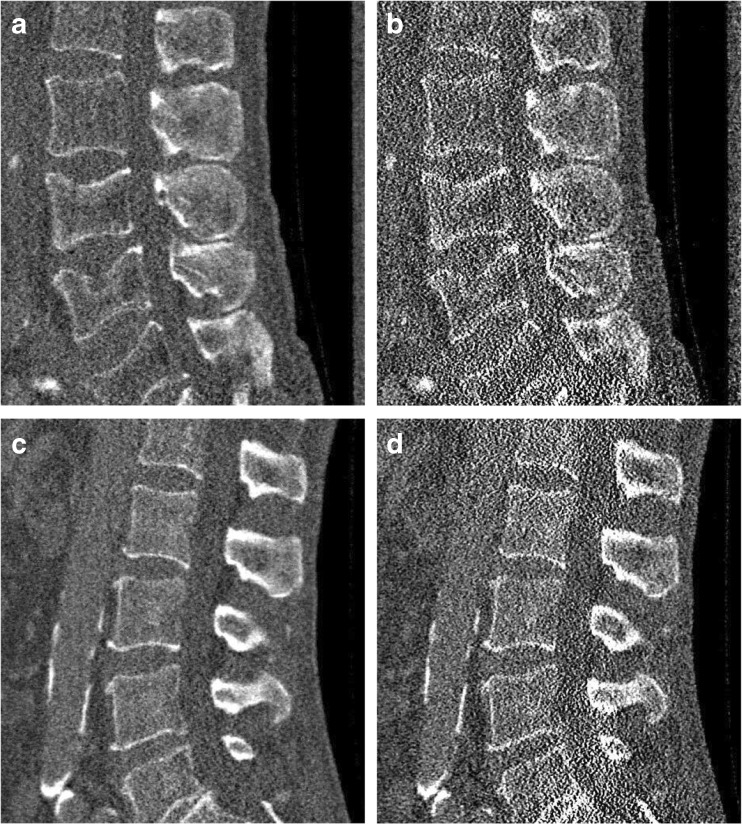
Representative sagittal reconstructions at 10% of the original dose level. The images depict the corresponding results for Fig. 1a and Fig. 1b. (**a**) and (**c**) represent the statistical iterative reconstruction (SIR) reconstructed image with 10% sparsely sampled projection. (**b**) and (**d**) show the SIR reconstructed images with the simulated 10% of the original tube current. Window level was 300 HU and width was 1,500 HU. Field of view was 180 × 153 mm^2^.

At 10% of the original dose, estimated SNR and CNR were five times better with sparse sampling than with simulated lower tube current images (Table 1). For sparsely sampled data, artefact patterns slowly became obvious when fewer projections were used. For simulated lower tube current images, streaking artefacts emerged with the decrease of tube current and image quality dropped rapidly. A representative matched subject pair with and without osteoporotic vertebral fracture at original dose is depicted in Fig. 1. Sparse sampling and simulated lower current images are depicted in Figs. 2, 3 and 4 and in the Appendix in Figs. A1 and A2.

**Fig. 5:**
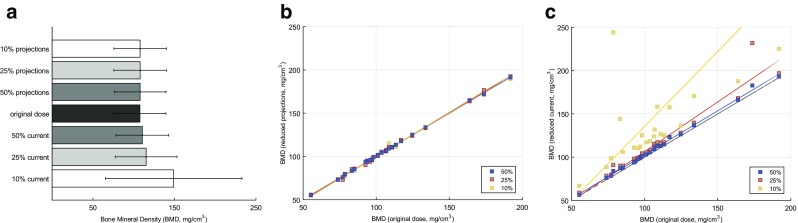
Bone mineral density (BMD) extracted from different image reconstructions. (**a**) The mean and standard deviations of all 24 subjects (mg/cm^3^). (**b**) The scatter plot of the measurements from sparse sampling images versus the original dose image. (**c**) The scatter plot of the measurements from the reduced tube current versus the original dose. The original dose is along the x-axis. Ultra-low-dose measurements at 50%, 25% and 10% are along the y-axis. The regression line is drawn in colour. The grey line depicts the centre line

**Fig. 6:**
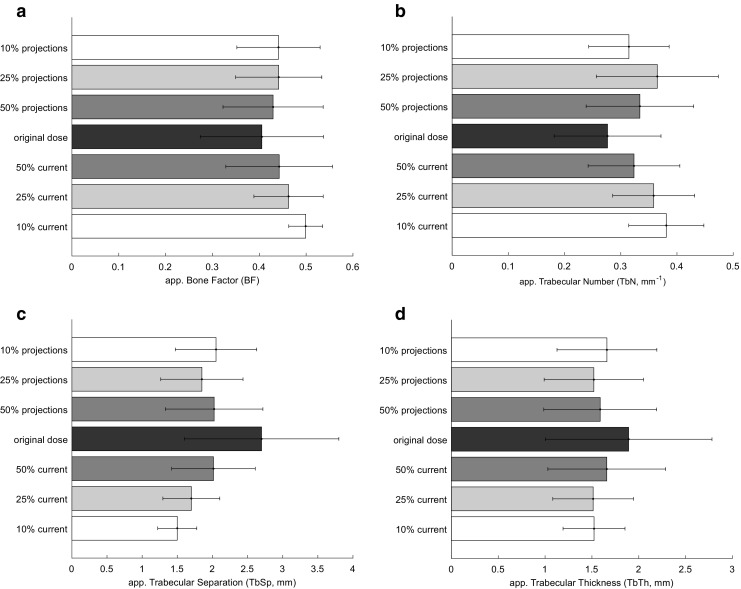
Trabecular parameters extracted from the different image reconstructions

We observed no significant change in BMD when analysing the reduced projections even at 10% sampling rate (*p* > 0.05), whereas lowering tube current to 10% resulted in on average 38% higher BMD values (Fig. 5). For all trabecular parameters, both sparse sampling and lowering tube current affected the measurements in various degrees. BF, TbN and FD tended to increase when dose was lowered, while TbSp and TbTh decreased. TbN and TbSp were most sensitive to the dose reduction (*p* < 0.001) around changes of 20–40% (Fig. 6). All changes in BMD and trabecular bone microstructure parameters from the different reconstructions are shown in Table 2 and in the Appendix in Fig. A3 and Table A1.

**Table 2:** Changes in bone mineral density (BMD) and trabecular bone microstructure parameters at reduced dose levels

**BMD**, mg/cm^-3^	Mean	SD	*p*-value	**App. BF**	Mean	SD	*p*-value
vs. original dose				vs. original dose			
Proj50	+0.216	0.797	.197	Proj50	+0.024	0.034	.002
Proj25	+0.145	1.547	.650	Proj25	+0.036	0.052	.003
Proj10	+0.233	1.978	.569	Proj10	+0.035	0.055	.004
Tube50	+2.900	1.863	.000	Tube50	+0.037	0.032	.000
Tube25	+7.546	11.040	.003	Tube25	+0.057	0.072	.001
Tube10	+41.32	67.131	.006	Tube10	+0.094	0.120	.001
**App. TbN**, mm^-1^	Mean	SD	*p*-value	**App. TbSp**, mm	Mean	SD	*p*-value
vs. original dose				vs. original dose			
Proj50	+0.058	0.025	.000	Proj50	-0.675	0.555	.000
Proj25	+0.089	0.066	.000	Proj25	-0.849	0.690	.000
Proj10	+0.038	0.047	.001	Proj10	-0.648	0.678	.000
Tube50	+0.046	0.027	.000	Tube50	-0.685	0.650	.000
Tube25	+0.082	0.048	.000	Tube25	-1.000	0.865	.000
Tube10	+0.104	0.091	.000	Tube10	-1.200	1.119	.000
**App. TbTh**, mm	Mean	SD	*p*-value	**FD**	Mean	SD	*p*-value
vs. original dose				vs. original dose			
Proj50	-0.304	0.402	.001	Proj50	+0.059	0.045	.000
Proj25	-0.372	0.520	.002	Proj25	+0.081	0.062	.000
Proj10	-0.232	0.527	.042	Proj10	+0.067	0.061	.000
Tube50	-0.234	0.377	.006	Tube50	+0.063	0.051	.000
Tube25	-0.379	0.642	.008	Tube25	+0.103	0.083	.000
Tube10	-0.370	0.797	.033	Tube10	+0.137	0.133	.000

Values are shown as compared to the original dose in all subjects (n=24) with respective p-values

Proj50, Proj25 and Proj10 indicate sparse sampling of 50%, 25% and 10% projection data, respectively

Tube50, Tube25 and Tube10 indicate simulation of 50%, 25% and 10% of the original tube current, respectively

Displayed means and standard deviations (SD) are given in absolute values

*App.* apparent, *BF* bone fraction, *BMD* bone mineral density, *FD* fractal dimension, *TbN* trabecular number, *TbSp* trabecular separation, *TbTh* trabecular thickness

For BMD, BF and TbTh, subjects without osteoporotic fractures still had greater values as compared to the matched subjects with osteoporotic fractures in both dose-reducing approaches. The two groups could still be differentiated, as differences were statistically significant (*p* < 0.05) even at 10% of the original dose level. For TbSp and FD, the differences between the two groups were significant only when the original dose was used, but was not significant when either data were sparsely sampled or tube current was reduced (*p* > 0.05). The differences in TbN between the two groups was not significant at any dose level. The mean, standard deviation, *p*-value and area under ROC curves of all parameters for differentiating the two groups are listed separately in Tables 3 and 4.

**Table 3:** Mean and standard deviation of bone mineral density (BMD) and trabecular bone microstructure

	Fracture		No fracture			Fracture		No fracture	
**BMD**	Mean	SD	Mean	SD	**App. BF**	Mean	SD	Mean	SD
SIR	90.236	17.455	125.228	33.868	SIR	0.311	0.095	0.500	0.160
Proj50	90.447	17.668	125.449	33.404	Proj50	0.356	0.082	0.503	0.127
Proj25	89.671	17.477	126.083	34.116	Proj25	0.378	0.074	0.505	0.107
Proj10	90.189	17.262	125.742	33.951	Proj10	0.375	0.072	0.507	0.103
Tube50	92.439	17.513	128.824	34.078	Tube50	0.362	0.083	0.523	0.138
Tube25	94.938	17.883	135.619	42.176	Tube25	0.406	0.065	0.519	0.082
Tube10	110.210	21.069	187.904	104.438	Tube10	0.472	0.035	0.526	0.037
**App. TbN**	Mean	SD	Mean	SD	**App. TbSp**	Mean	SD	Mean	SD
SIR	0.284	0.108	0.269	0.080	SIR	3.127	1.178	2.272	1.015
Proj50	0.352	0.106	0.316	0.084	Proj50	2.148	0.605	1.901	0.773
Proj25	0.377	0.108	0.353	0.109	Proj25	1.896	0.495	1.804	0.664
Proj10	0.331	0.074	0.298	0.069	Proj10	2.145	0.483	1.958	0.662
Tube50	0.341	0.091	0.306	0.071	Tube50	2.158	0.515	1.871	0.665
Tube25	0.378	0.080	0.339	0.065	Tube25	1.752	0.356	1.647	0.448
Tube10	0.412	0.073	0.350	0.060	Tube10	1.416	0.256	1.583	0.298
**App. TbTh**	Mean	SD	Mean	SD	**FD**	Mean	SD	Mean	SD
SIR	1.301	0.609	2.483	1.100	SIR	1.119	0.163	1.225	0.147
Proj50	1.171	0.477	2.006	0.706	Proj50	1.207	0.145	1.254	0.134
Proj25	1.165	0.477	1.876	0.579	Proj25	1.241	0.146	1.264	0.126
Proj10	1.282	0.441	2.038	0.611	Proj10	1.219	0.140	1.259	0.135
Tube50	1.234	0.510	2.083	0.728	Tube50	1.211	0.146	1.259	0.126
Tube25	1.218	0.369	1.808	0.485	Tube25	1.269	0.142	1.282	0.112
Tube10	1.284	0.308	1.761	0.352	Tube10	1.331	0.145	1.287	0.110

Parameters are shown as matched groups with (n=12) and without vertebral fracture (n=12) for the different dose levels

SIR indicates iterative reconstruction of the original dose

Proj50, Proj25 and Proj10 indicate sparse sampling of 50%, 25% and 10% projection data, respectively

Tube50, Tube25 and Tube10 indicate simulation of 50%, 25% and 10% of the original tube current, respectively

*App.* apparent, *BF* bone fraction, *BMD* bone mineral density, *FD* fractal dimension, *TbN* trabecular number, *TbSp* trabecular separation, *TbTh* trabecular thickness

**Table 4:** *P*-values and area under the receiver-operating characteristic (ROC) curve for the fracture and no-fracture groups, observed at different dose levels

**BMD**	*p*-value	ROC	**App. BF**	*p*-value	ROC
SIR	.002*	.875	SIR	.002*	.861
Proj50	.002*	.875	Proj50	.002*	.840
Proj25	.002*	.875	Proj25	.002*	.833
Proj10	.002*	.868	Proj10	.001*	.854
Tube50	.002*	.875	Tube50	.003*	.878
Tube25	.002*	.882	Tube25	.001*	.868
Tube10	.023*	.896	Tube10	.001*	.878
**App. TbN**	*p*-value	ROC	**App. TbSp**	*p*-value	ROC
SIR	.699	.458	SIR	.028*	.319
Proj50	.371	.375	Proj50	.279	.396
Proj25	.601	.417	Proj25	.640	.458
Proj10	.285	.368	Proj10	.347	.417
Tube50	.351	.372	Tube50	.201	.382
Tube25	.253	.361	Tube25	.459	.424
Tube10	.033*	.236	Tube10	.079	.656
**App. TbTh**	*p*-value	ROC	**FD**	*p*-value	ROC
SIR	.004*	.861	SIR	.087	.694
Proj50	.003*	.854	Proj50	.385	.618
Proj25	.005*	.861	Proj25	.650	.587
Proj10	.004*	.896	Proj10	.447	.597
Tube50	.006*	.868	Tube50	.378	.618
Tube25	.010*	.875	Tube25	.808	.556
Tube10	.002*	.833	Tube10	.381	.417

Parameters are shown as matched groups with (n=12) and without vertebral fracture (n=12) for the different dose levels

ROC denotes area under the ROC curve

SIR indicates iterative reconstruction of the original dose

Proj50, Proj25 and Proj10 indicate sparse sampling of 50%, 25% and 10% projection data

Tube50, Tube25 and Tube10 indicate simulation of 50%, 25% and 10% of the original tube current

* indicates p-values with statistically significant differences between the two groups (p<0.05)

*App.* apparent, *BF* bone fraction, *BMD* bone mineral density, *FD* fractal dimension, *TbN* trabecular number, *TbSp* trabecular separation, *TbTh* trabecular thickness

## Discussion

To the best of our knowledge, this is the very first study of in-vivo data comparing reduced tube current and projection angles with statistical iterative reconstruction with regard to BMD and trabecular bone microstructure analysis. Our results demonstrated that it is computationally possible to assess the bone microstructure quantitatively with half or less of the dose in non-dedicated routine MDCT. BMD, BF and TbTh, were robust to dose changes and still differentiated between subjects with and without osteoporotic vertebral fractures, while TbSp and FD were dose-sensitive.

For sparse sampling, we observed that vertebral BMD did not change on a statistically significant level when less projection angles (down to only 10%) were used. This may allow BMD measurements with much lower radiation exposure in the future. However, it is not yet possible to apply sparse sampling to lower radiation exposure at commercial CT scanners. In current systems, the X-ray source is constantly delivering X-rays during the entire examination. However, precise and fast grid-switching units are reported to be introduced to control the X-ray source on and off in the future. On the other hand, tube current reduction, or modulation, is relatively easy to realize and has been intensively studied [10]. Current detectors are more suspended to electronic noise, especially when the tube current is lowered. Electronic noise can destroy the signal or can cause a bias to R1.4quantitative values. For this reason, we observed significant changes in absolute BMD values when tube current was lowered, compared to the data from sparse sampling. This suggests that values from sparse sampling seem to be more robust than values from lower tube currents for BMD measurement. However, the computed parameters at ultra-low-dose levels from both approaches still adequately differentiated subjects with and without osteoporotic fractures.

In our study, the mean CTDI_vol_ value was 7.5 mGy (max. 13.7 mGy, min. 5.1 mGy, among abdomen scans) in the original scans. The approximated effective dose was about 2.6 mSv per person (estimation of T12–L5 coverage [28]). Our results showed that further dose reduction was still adequate to approximate quantitative bone parameters; this was actually much lower than previously reported (1.5 mSv for L1 and L2 only [29]).

We used statistical iterative reconstruction (SIR) in this study. Compared to the reconstruction process based on traditional methods like FBP, an iterative-based algorithm is considered more suitable handling noise and streaking artefacts, because it integrates physics modeling, providing better performances for missing data, irregular sampling and tube current reduction [30]. A higher level of the modelling provides greater image quality improvement, but also at a greater price of computational complexity. Because the image quality generated by traditional algorithms is still acceptable for most diagnostic purposes, additional algorithms with higher modelling complexity are often used in academics and avoided by manufacturers for clinical routine [31]. However, with the vast development of graphic units and computational power, SIR will become much more widely available [32]. The image noise in SIR is mainly handled with a regularization term. A previous study [23] investigated the effect of different models of regularization on trabecular bone microstructure at the spine in-vitro. These investigators observed that a minimum of regularization is best suited for quantitative bone microstructure analysis. For this reason, we also used a low regularization term in this study.

Admittedly our study had limitations. Firstly, the number of the investigated subjects was relatively small due to the pilot character of this study. Secondly, the decisions of osteoporotic or control subjects were determined merely by the presentation of actual vertebral fractures. Thirdly, MDCT imaging was performed with application of an intravenous contrast agent due to the clinical indication of the examinations, which affects quantitative bone measurements. However, a BMD conversion equation was applied as reported previously [24]. Lastly, low-dose scans in this study were simulated retrospectively and were not acquired prospectively due to radiation protection regulations. However, the validity of the low-dose simulation tool has been demonstrated previously both by the industrial manufacturers’ laboratory [18] and clinical research institution [19]. Further studies have to be performed in the future to validate the potential of dose reduction approaches for CT-based osteoporosis diagnostics and therapy monitoring.

In conclusion, we investigated the effect of sparse sampling and simulated lower tube current in non-dedicated MDCT scans combined with SIR on BMD and quantitative bone microstructure assessment. Our findings indicate that BMD and trabecular bone microstructure are still assessable at ultra-low dose levels. BMD, apparent bone factor and trabecular thickness were able to differentiate between subjects with and without osteoporotic fractures based on both approaches for dose reduction, sparse sampling and lower tube currents. This suggests that fracture risk prediction with low-dose protocols is feasible. However, absolute parameter values at reduced dose levels significantly differed from original values. BMD measurements derived from sparse sampling showing less changes compared to values acquired with lower tube currents, suggesting sparse sampling to be a more robust dose reduction approach for BMD measurements. These changes in parameters should be considered for future studies and clinical use.

## Electronic supplementary material

Below is the link to the electronic supplementary material.

ESM 1 (DOCX 1716 kb)

## Abbreviations

app: Apparent
BF: Bone fraction
BMD: Bone mineral density
CNR: Contrast-to-noise ratio
DXA: Dual-energy X-ray absorptiometry
FD: Fractal dimension
MDCT: Multidetector computed tomography
PPR: Projections per full rotation
QCT: Quantitative computed tomography
ROC: Receiver operating characteristic
ROI: Region of interest
SIR: Statistical iterative reconstruction
SNR: Signal-to-noise ratio
TbN: Trabecular number
TbSp: Trabecular separation
TbTh: Trabecular thickness
