# Supplementary material for: Is multidetector CT-based bone mineral density and quantitative bone microstructure assessment at the spine still feasible using ultra-low tube current and sparse sampling?
Source: Eur Radiol. 2017 Jun 21;27(12):5261–71. doi: 10.1007/s00330-017-4904-y (PMC5674130; doi:10.1007/s00330-017-4904-y)
Supplement: Supplementary file 1 — (DOCX 1716 kb) [file 330_2017_4904_MOESM1_ESM.docx]

**[Fig.1]** Representative sagittal reconstructions of the lumbar spine (L1-L5) of in-vivo spine MDCT data. Both left columns depict a subject with fracture (1); both right columns display the matched healthy subject with regard to age and gender (2). (a) represent the SIR reconstructed image with original dose: 120 kV, 107 mAs(1) and 114 mAs(2), (exact tube current was modulated) . (b)(d)(f) show the SIR reconstructed images with 50%, 25% and 10% reduced projection. (c)(e)(g) show the SIR reconstructed images with the simulated 50%, 25% and 10% of the original tube current. Window level was 300 HU and width was 1500 HU. Field of view was 180x153 mm^2^.

| 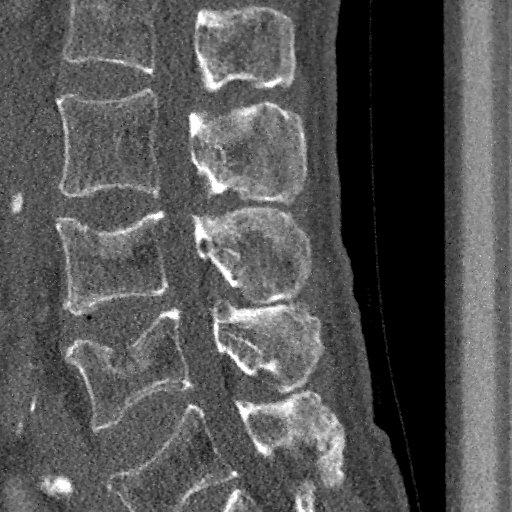 | | 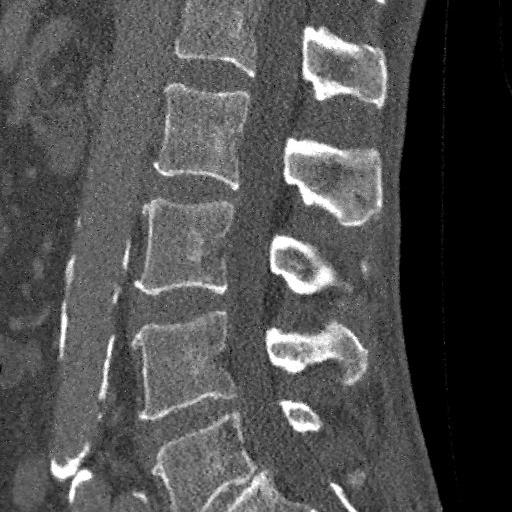 | |
| --- | --- | --- | --- |
| (a1) | | (a2) | |
| 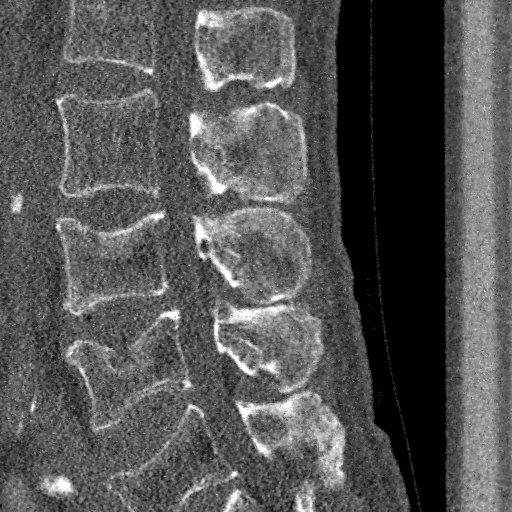 | 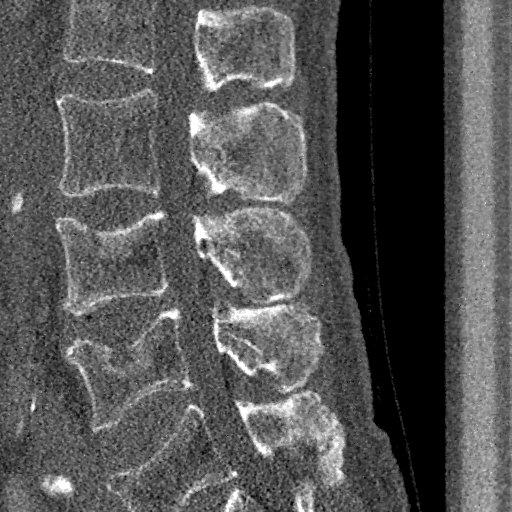 | 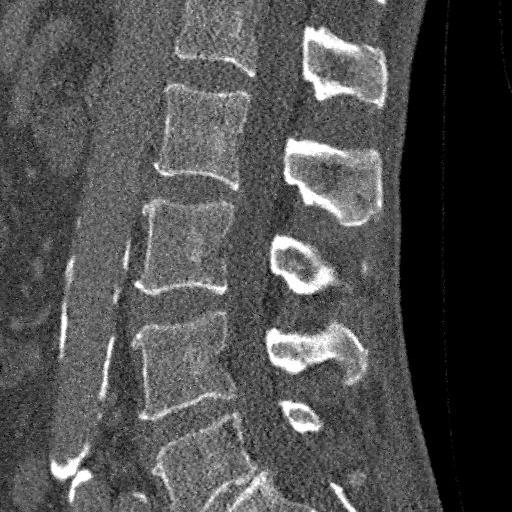 | 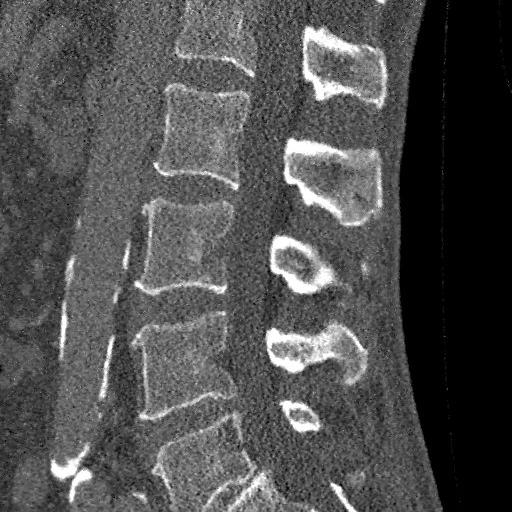 |
| (b1) | (c1) | (b2) | (c2) |
| 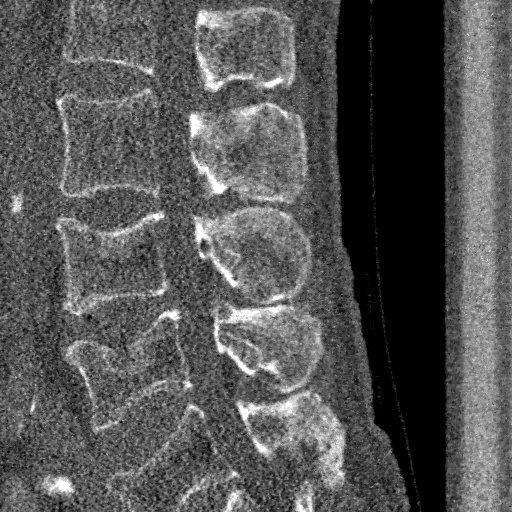 | 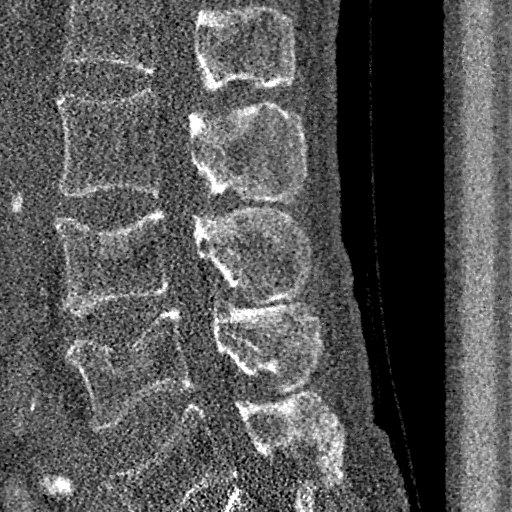 | 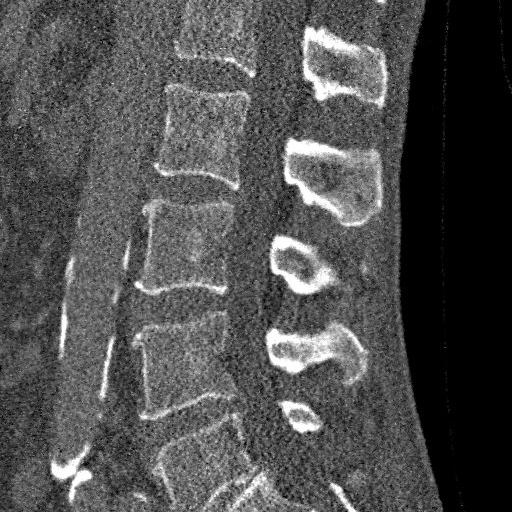 | 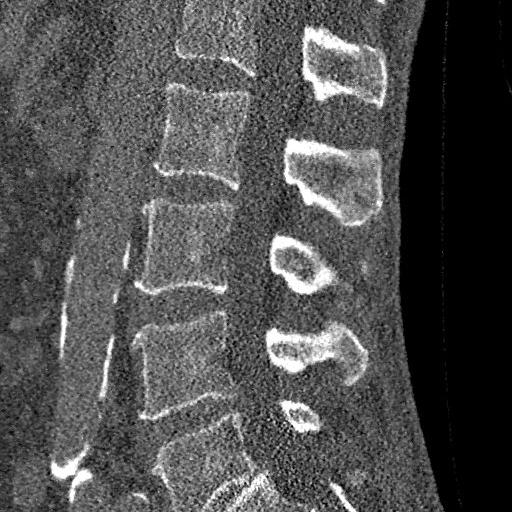 |
| (d1) | (e1) | (d2) | (e2) |
| 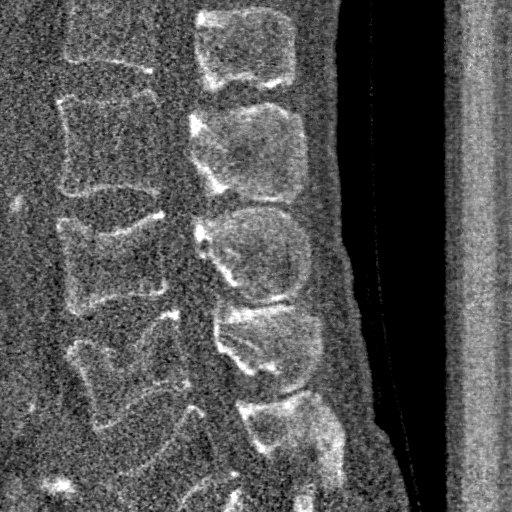 | 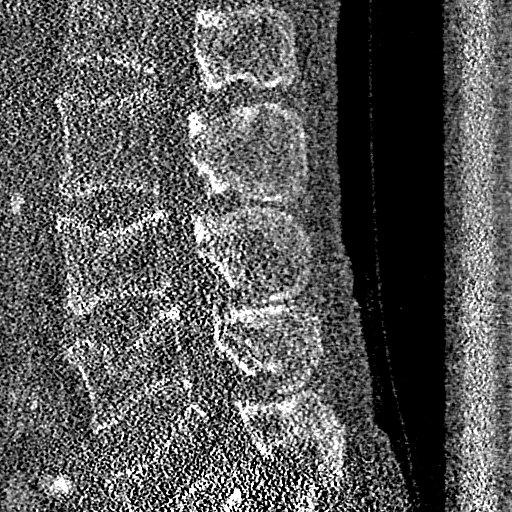 | 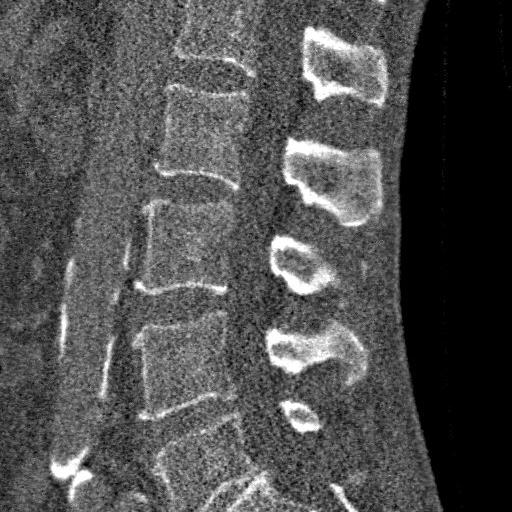 | 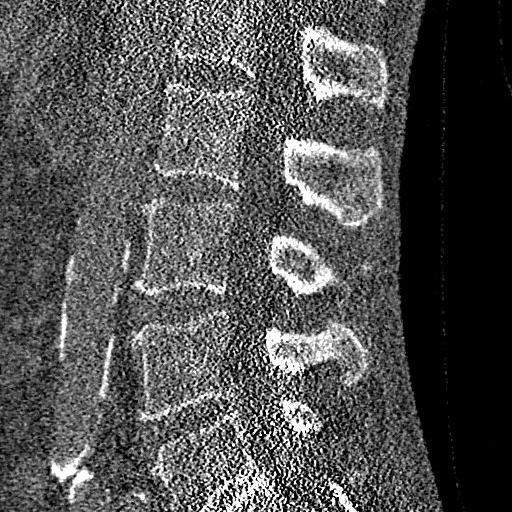 |
| (f1) | (g1) | (f2) | (g2) |

**[Fig.2]** Representative axial reconstructions of in-vivo spine MDCT data. Both left columns depict a subject with fracture (1); both right columns display the matched healthy subject with regard to age and gender (2). (a) show the SIR reconstructed image with original dose: 120kV, 107 mAs(1) and 114 mAs(2), (exact tube current was modulated). (b)(d)(f) show the SIR reconstructed images with 50%, 25% and 10% reduced projection. (c)(e)(g) show the SIR reconstructed images with simulated 50%, 25% and 10% of the original tube current. Axial slices show a section across lumbar vertebra L2. Window level was 300 HU and width was 1500 HU. Field of view was 156x156 mm^2^.

| 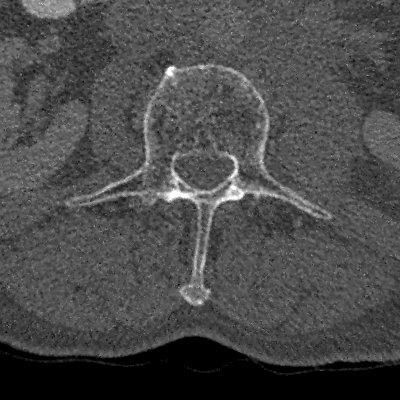 | | 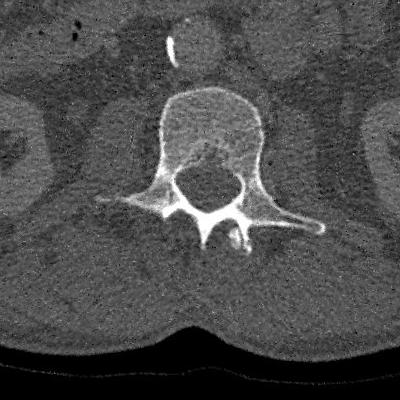 | |
| --- | --- | --- | --- |
| (a1) | | (a2) | |
| 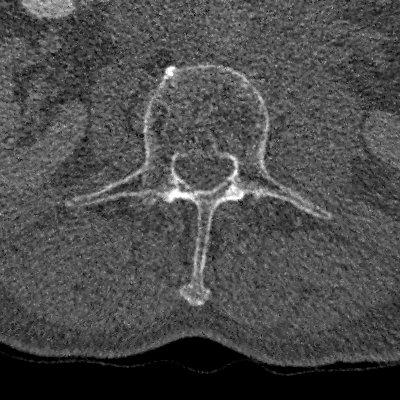 | 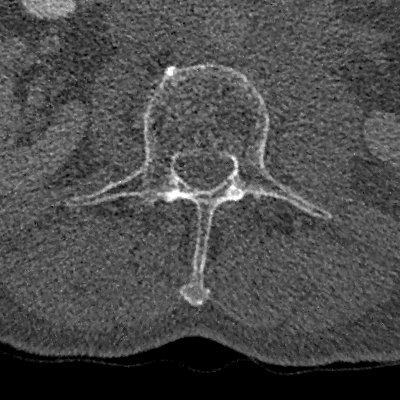 | 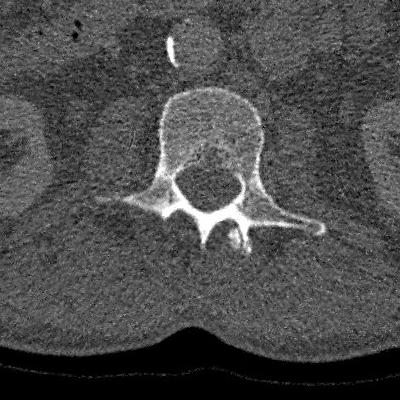 | 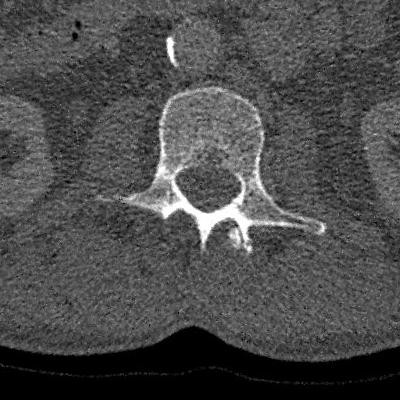 |
| (b1) | (c1) | (b2) | (c2) |
| 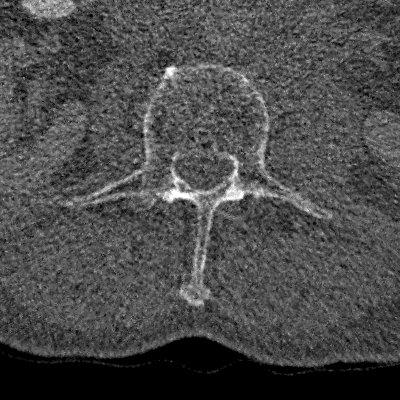 | 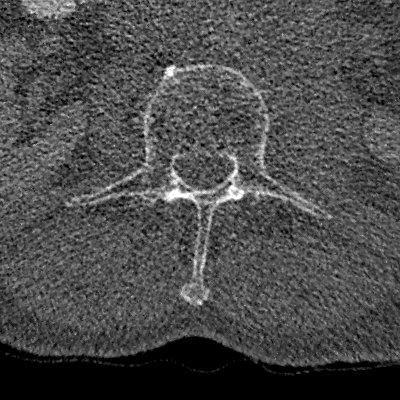 | 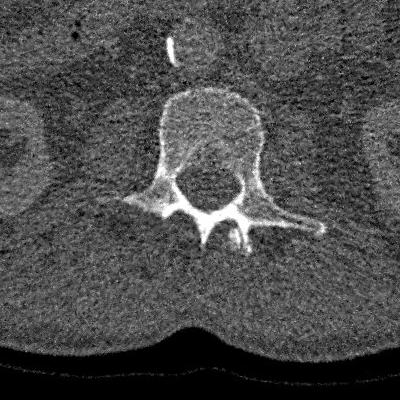 | 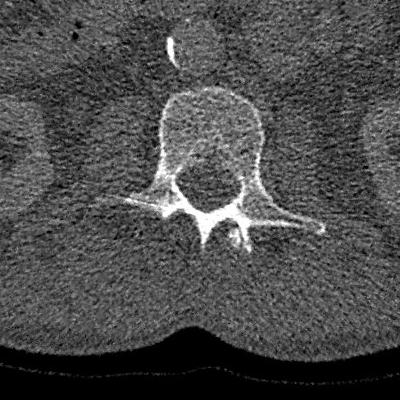 |
| (d1) | (e1) | (d2) | (e2) |
| 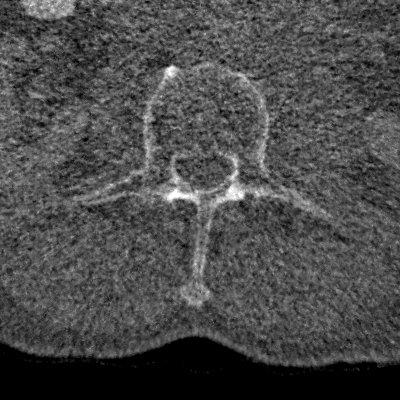 | 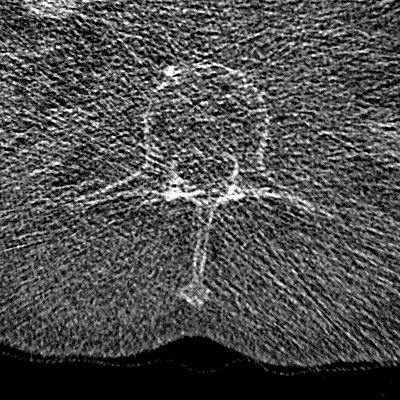 | 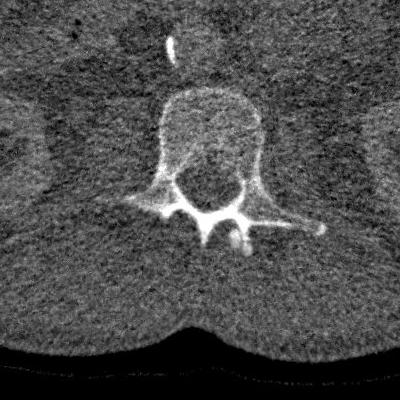 | 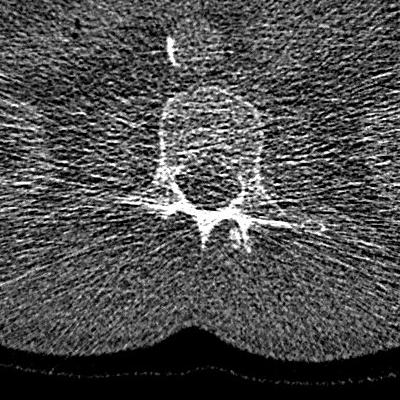 |
| (f1) | (g1) | (f2) | (g2) |

**[Fig.3]** BMD and trabecular bone microstructure parameters extracted from the different image reconstructions. From top to bottom: Bone Mineral Density(BMD), App. Bone Fraction (BF), App. Trabecular Number (TbN), App. Trabecular Separation (TbSp), App. Trabecular Thickness (TbTh) and Fractal Dimension(FD). The first column shows the mean and standard deviations of all 24 subjects. The second column illustrates the scatter plot of the measurements of reduced projections images versus the original dose image. The third column illustrates the scatter plot of the measurements of reduced tube current versus the original dose. Regression line is drawn in color. The gray line depicts the center line.

| 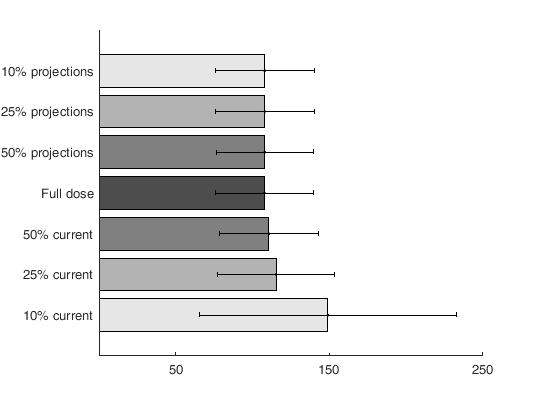 | 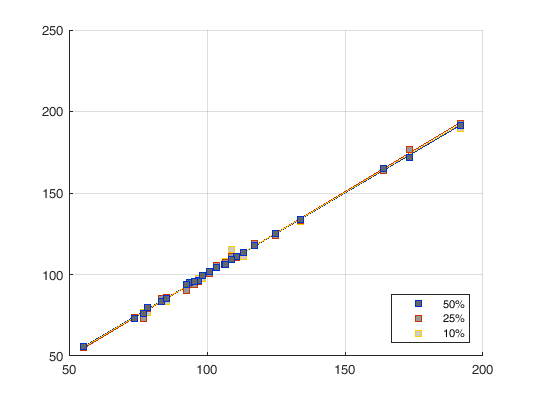 | 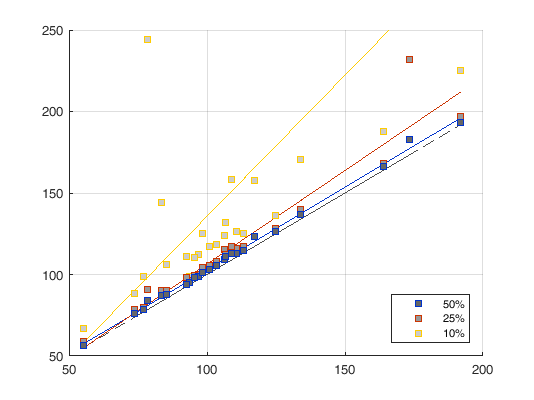 |
| --- | --- | --- |
| (a) Bone Mineral Density (BMD), mg/cm^3^ | (b) BMD, original dose (x-axis) vs. reduced projections (y-axis) | (c) BMD, original dose (x-axis) vs. reduced current (y-axis) |
| 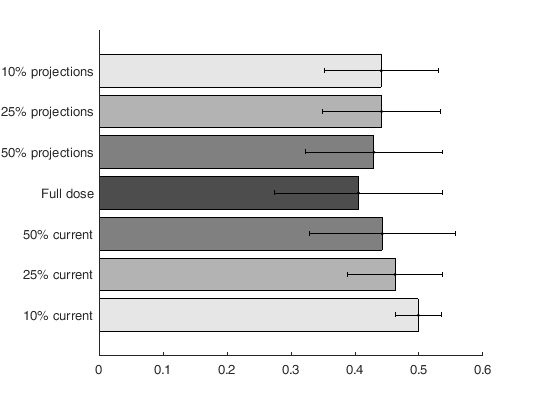 | 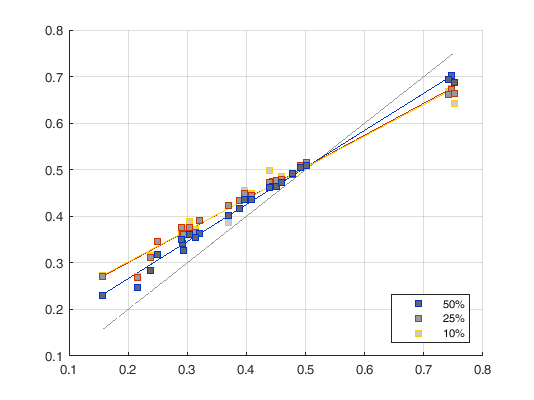 | 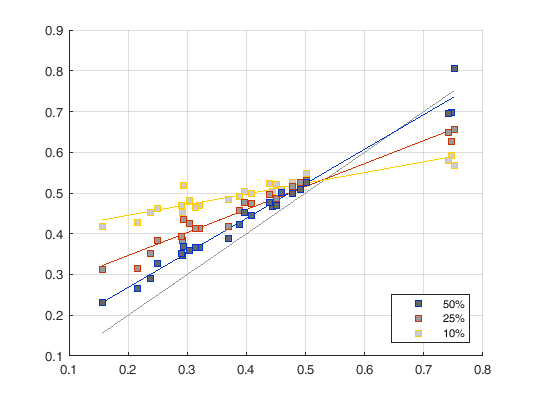 |
| (d) App. Bone Volume / Total Volume (BF) | (e) App. BF, original dose (x-axis) vs. reduced projections (y-axis) | (f) App. BF, original dose (x-axis) vs. reduced current (y-axis) |
| 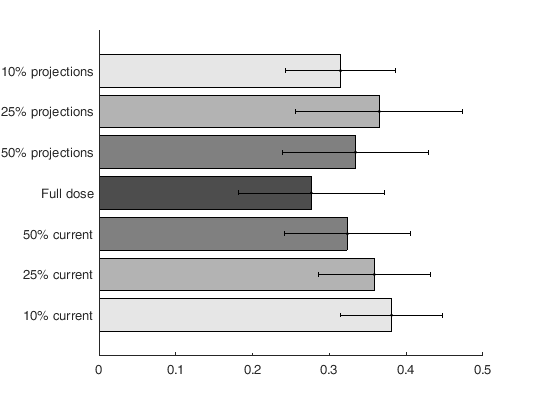 | 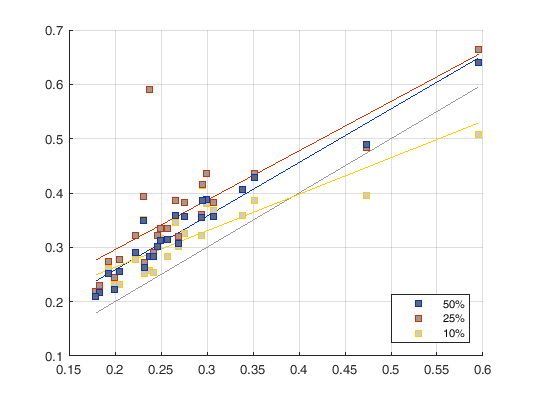 | 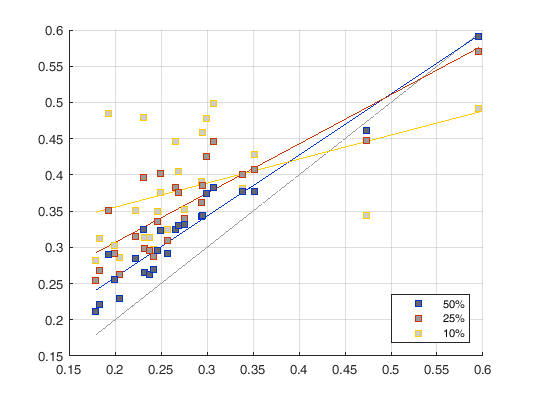 |
| (g) App. Trabecular Number (TbN), mm^-1^ | (h) App. TbN, original dose (x-axis) vs. reduced projections (y-axis) | (i) App. TbN, original dose (x-axis) vs. reduced current (y-axis) |
| 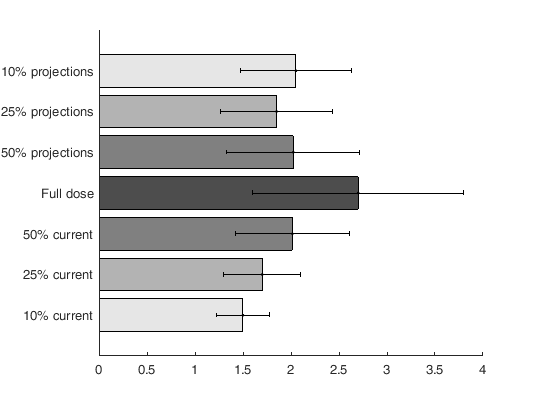 | 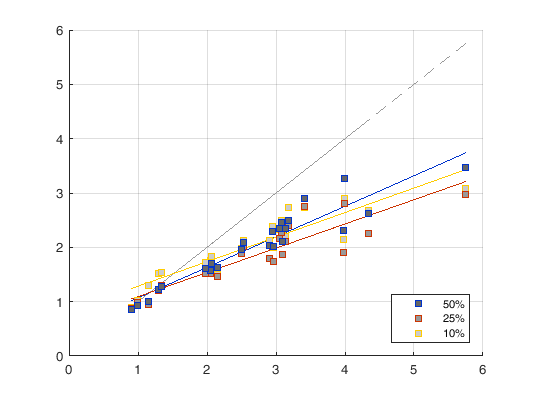 | 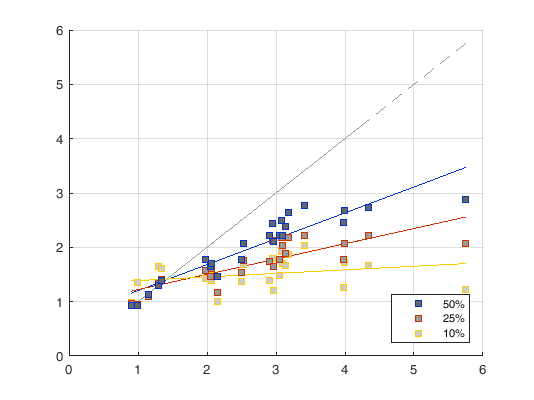 |
| (j) App. Trabecular Separation (TbSp), mm | (k) App. TbSp, original dose (x-axis) vs. reduced projections (y-axis) | (l) App. TbSp, original dose (x-axis) vs. reduced current (y-axis) |
| 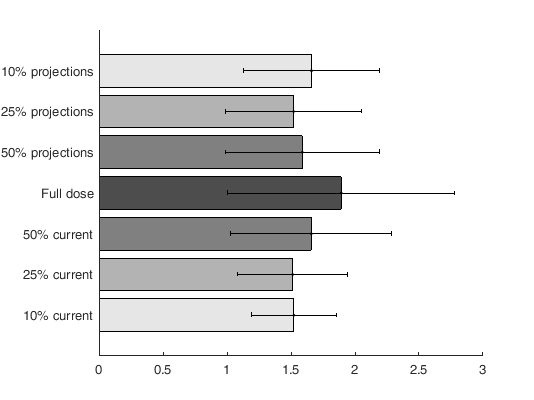 | 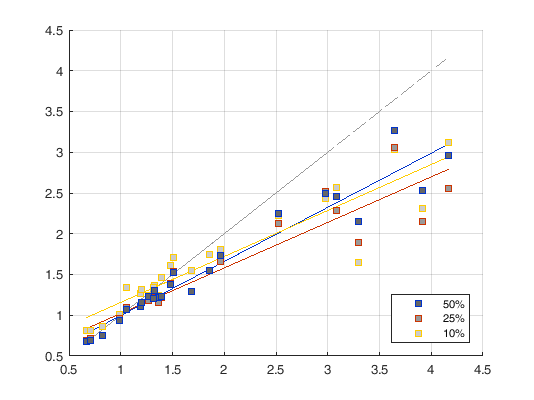 | 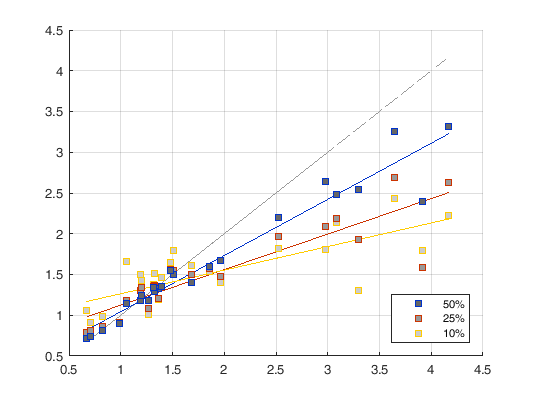 |
| (m) App. Trabecular Thickness (TbTh), mm | (o) App. TbTh, original dose (x-axis) vs. reduced projections (y-axis) | (p) App. TbTh, original dose (x-axis) vs. reduced current (y-axis) |
| 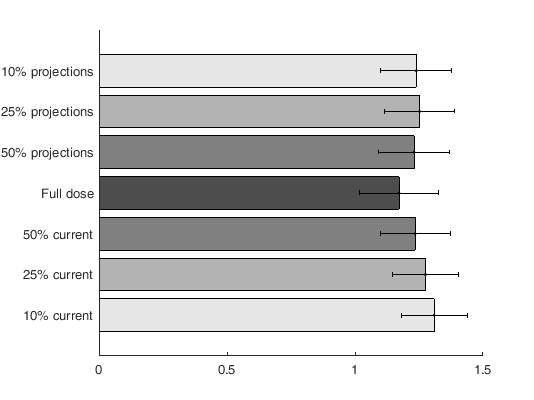 | 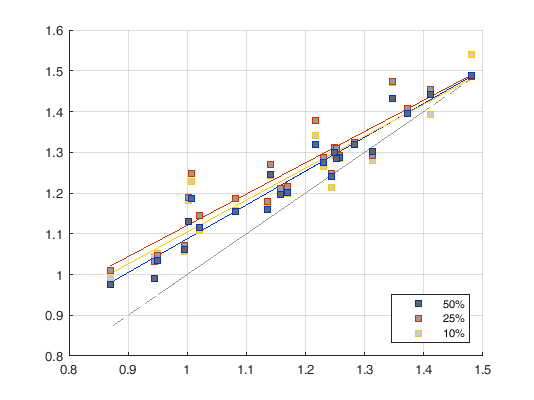 | 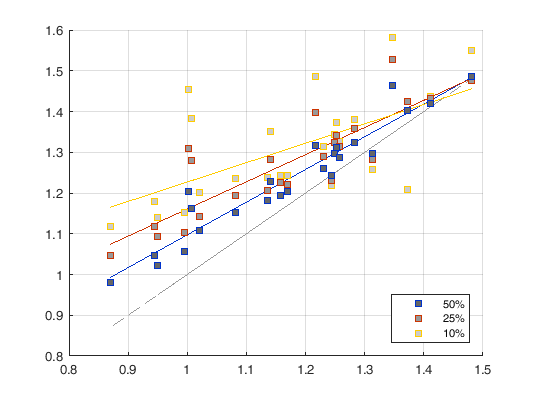 |
| (q) Fractal Dimension | (r) FD, original dose (x-axis) vs. reduced projections (y-axis) | (s) FD, original dose (x-axis) vs. reduced current (y-axis) |

**[Tab.1]** Correlation coefficient r and p-value of sparse sampling (reduced projections) and simulated lower tube current (reduced current) versus the original dose in all subjects (n=24).

|  | Reduced projection | | Reduced current | |  |  | Reduced projection | | Reduced current | |
| --- | --- | --- | --- | --- | --- | --- | --- | --- | --- | --- |
| **BMD** | r | p-value | r | p-value |  | **App. BF** | r | p-value | r | p-value |
| 50% | 1.000 | .000 | 0.995 | .000 |  | 50% | 0.997 | .000 | 0.989 | .000 |
| 25% | 0.999 | .000 | 0.965 | .000 |  | 25% | 0.994 | .000 | 0.982 | .000 |
| 10% | 0.998 | .000 | 0.659 | .000 |  | 10% | 0.988 | .000 | 0.941 | .000 |
|  |  |  |  |  |  |  |  |  |  |  |
| **App. TbN** | r | p-value | r | p-value |  | **App. TbSp** | r | p-value | r | p-value |
| 50% | 0.965 | .000 | 0.962 | .000 |  | 50% | 0.946 | .000 | 0.923 | .000 |
| 25% | 0.793 | .000 | 0.859 | .000 |  | 25% | 0.901 | .000 | 0.818 | .000 |
| 10% | 0.869 | .000 | 0.424 | .039 |  | 10% | 0.913 | .000 | 0.268 | .205 |
|  |  |  |  |  |  |  |  |  |  |  |
| **App. TbTh** | r | p-value | r | p-value |  | **FD** | r | p-value | r | p-value |
| 50% | 0.966 | .000 | 0.970 | .000 |  | 50% | 0.966 | .000 | 0.956 | .000 |
| 25% | 0.933 | .000 | 0.891 | .000 |  | 25% | 0.928 | .000 | 0.861 | .000 |
| 10% | 0.920 | .000 | 0.757 | .000 |  | 10% | 0.930 | .000 | 0.599 | .002 |
